# Disabled‐1 is down‐regulated in clinical breast cancer and regulates cell apoptosis through NF‐κB/Bcl‐2/caspase‐9

**DOI:** 10.1111/jcmm.14047

**Published:** 2018-11-28

**Authors:** Rang‐Juan Cao, Kai Li, Wan‐Ying Xing, Shuang Du, Qiang Li, Xiao‐Juan Zhu, Shu‐Sen Cui

**Affiliations:** ^1^ Department of Hand Surgery China‐Japan Union Hospital of Jilin University Changchun China; ^2^ Department of Anesthesia China‐Japan Union Hospital of Jilin University Changchun China; ^3^ Department of Breast Surgery China‐Japan Union Hospital of Jilin University Changchun China; ^4^ Key Laboratory of Molecular Epigenetics of Ministry of Education Institute of Genetics and Cytology Northeast Normal University Changchun China

**Keywords:** breast cancer, cell apoptosis, clinical prognosis, disabled‐1, NF‐κB

## Abstract

Disabled‐1 (Dab1) is best known as an adaptor protein regulating neuron migration and lamination during development. However, the exact function of Dab1 in breast cancer is unknown. In this study, we examined the expression of Dab1 in 38 breast cancer paraffin sections, as well as 60 paired frozen breast cancer and their adjacent tissues. Our results showed that Dab1 was reduced in breast cancer, and its compromised expression correlated with triple negative breast cancer phenotype, poor differentiation, as well as lymph node metastasis. Functional analysis in breast cancer cell lines demonstrated that Dab1 promoted cell apoptosis, which, at least partially, depended on its regulation of NF‐κB/Bcl‐2/caspase‐9 pathway. Our study strongly suggests that *Dab1* may be a potential tumour suppressor gene in breast cancer.

## 1 INTRODUCTION

Breast cancer, based on the cancer statistics of 2017, is still the most common cancer which accounts for 30% of all new cancer diagnoses.^1^ Clinically, tumour stage, histological grade and the status of lymph node have been used as indicators of breast cancer progression, treatment response and prognosis.^2^ However, it is still the second most mortality cancer, accounting for 14% of all cancer‐caused deaths in women.^1^ Therefore, identification of novel tumour therapeutic targets is still important for early detection and follow‐up treatments.

*Dab1* gene encodes a 555 amino acids protein with an N‐terminal PTB domain, a region containing important Tyr residues, and a C‐terminal region.^3^ It is a well‐documented intracellular messenger of reelin signalling pathway, and extremely important for neuron migration and lamination in developing brain.^4^ Common fragile sites (CFS) are large chromosomal regions of instability existing in all individuals, and proteins coded by genes with CFS are usually demonstrated to be tumour suppressors.^5^ A study reported that *Dab1* gene was a CFS gene and showed reduced expression in cancer‐derived cell lines or primary tumours of ovary, prostate, breast, endometrium and brain.^6^ However, the role of *Dab1* in clinical and whether it functions as a suppressor gene, like other CFS genes, in human breast cancer are unknown.

Here, we found *Dab1* was down‐regulated in breast cancer and displayed a negative correlation with triple negative breast cancer phenotype, poor differentiation, lymph node metastasis and other prognosis parameters. Functional analysis revealed a pro‐apoptotic role of Dab1 via the inhibition of NF‐κB subunit p65, down‐regulation of pro‐survival protein Bcl2 and up‐regulation of pro‐apoptosis proteins Bid and Bax. These data suggested that *Dab1* might function as a tumour suppressor gene down‐regulated in breast cancer.

## 2 MATERIALS AND METHODS

Antibodies and reagents, as well as experimental details, were described in Supplementary Materials and Methods.

Shortly, clinical specimens were collected in accordance with the ethical standards of the guidelines of the ethics committee of China‐Japan Union Hospital of Jilin University and the World Medical Association Declaration of Helsinki. Informed consent was obtained from all the patients recruited randomly in this study. The 38 paraffin sections of patients who went modified radical mastectomy without any pre‐operative treatment, used in immunohistochemistry staining, were obtained from Department of Pathology in China‐Japan Union Hospital of Jilin University between January 2016 and November 2016 (Table S1). The 60 frozen specimens, used for protein and mRNA extraction, were collected from patients receiving surgery with non‐preoperative therapy between January 2017 and July 2017 (Table S2). Fresh tumour tissues and adjacent non‐cancerous tissues were collected during surgery, snap‐frozen in liquid nitrogen and stored at −80°C before use. All the pathological results of specimens were confirmed by histopathological analysis, and adjacent non‐cancerous tissues were defined as 3 cm away from the tumour margin and must not contain tumour cells. Samples were randomly chosen and sequence numbers refer to the patient numbers in Supplementary Tables. The expression of Dab1 in breast cancer tissues or cell lines was assessed by immunohistochemistry staining or Western blot or real‐time PCR. MCF‐10A, MCF‐7, BT‐549, MDA‐MB‐231 and 293T were obtained from the American Type Culture Collection. Target shRNAs designed with BLOCK‐iT™ RNAi Designer and targeting the region encompassing nucleotides 1221‐1239 (shDab1^1221^) or 1515‐1533 (shDab1^1515^) were cloned into lentiviral plasmid PLL3.7. The *Dab1* expressing plasmid was cloned into a lentivirus expressing vector pWPXLd. Cell growth was analysed by CCK8, while cell apoptosis was labelled by Annexin V‐FITC/PI kit or Annexin V‐APC/7‐AAD kit. Statistical analysis was performed with SPSS17.0. Kruskal–Wallis test was used for the comparisons of three or more groups, and non‐parametric Mann–Whitney *U* test was used for the comparisons between two groups. All the graphics were performed with GraphPad Prism 5.0. *P*‐value <0.05 was considered as significant.

## 3 RESULTS AND DISCUSSION

### 3.1 Dab1 was down‐regulated in breast cancer tissues and negatively correlated with poor prognosis factors

**Figure 1:**
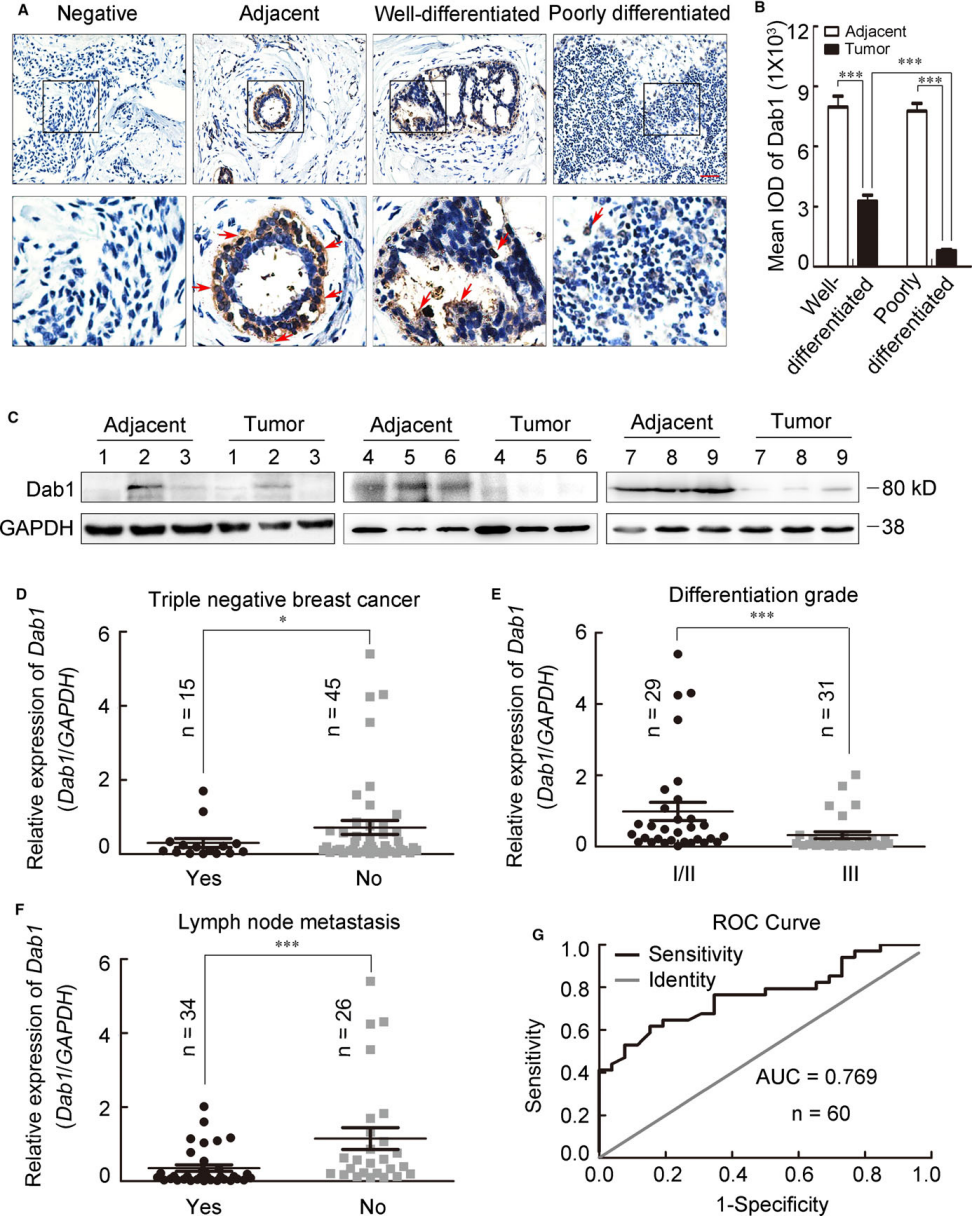
The expression of Dab1 was decreased in breast cancer tissues and negatively correlated with poor prognosis factors. (A) Representative images of immunohistochemistry staining in different tissues. The arrows indicated Dab1 positive cells (brown signal). Boxed areas in the upper panel were magnified and shown in the lower panel. Scale bar = 500 μm. (B) Statistical analysis of the mean IOD of well‐differentiated and poorly differentiated breast cancer tissues, as well as their corresponding adjacent tissues, ***P < 0.001. (C) Western blot analysis of the expression of Dab1 in nine paired clinical breast cancer tissues and their adjacent tissues chose randomly. GAPDH was an internal control. (D‐F) The statistical analysis between relative expression of *Dab1* and triple negative breast cancer (0.301 ± 0.124 vs 0.833 ± 0.185, *P* = 0.027), differentiation grade (0.529 ± 0.098 vs 1.419 ± 0.255, *P* < 0.001) or lymph node metastasis (0.355 ± 0.088 vs 1.152 ± 0.295, *P* < 0.001). (G) ROC curve for *Dab1*. AUC was 0.769, and sensitivity and specificity were 0.846 and 0.618. The data were shown as mean ± *SEM*. For all statistics analysis in this figure, *P < 0.05; ***P < 0.001

The expression pattern of Dab1 in clinical paraffin tissues (n = 38) was firstly assessed by immunohistochemistry staining. Results showed that Dab1 (arrows) was abundant in mammary ductal epithelial cells of adjacent non‐cancerous tissues, reduced in well‐differentiated breast cancer tissues, but almost undetectable in poorly differentiated breast cancer tissues (Figure 1A). Mean integral optical density (IOD) was analysed by Image‐Pro Plus and poorly differentiated tissues exhibited significantly reduced expression of Dab1 (Figure 1B). To quantify the reduced expression, Dab1 in an additional nine pairs of frozen breast cancer and adjacent tissues were analysed by Western blot and, consistently, reduced in eight of the nine patients (88.89%, Figure 1C). Real‐time PCR was carried out in breast cancer specimens from 60 patients to examine the *Dab1* mRNA profile. Among these, 46 patients (76.67%) exhibited reduced expressions of *Dab1* in tumour tissues compared to their paired adjacent tissues (Figure S1A, *Dab1* in adjacent non‐malignant tissue was normalized to 1). The relationship between *Dab1* and clinic‐pathological parameters was further analysed. Oestrogen receptor (ER) and progesterone receptor (PR) negative tumours were disabled to respond to endocrine agents correlated with a worse clinical outcome.^7^ Reduced expression of *Dab1* was observed in ER negative patients compared to positive ones (0.389 ± 0.098 vs 1.033 ± 0.270, *P* = 0.01), as well as PR negative patients to positive ones (0.378 ± 0.092 vs 1.033 ± 0.270, *P* = 0.007). Triple negative breast cancer, negative reaction with ER, PR and human epidermal growth factor receptor 2 (HER2), is a high‐risk subtype with no special therapy target protein, showed a sharply reduced expression of *Dab1* (Figure 1D, 0.301 ± 0.124 vs 0.833 ± 0.185, *P* = 0.027).

The degree of histological differentiation is one of the best established prognostic factors.^8^ Breast cancer patients are separated into three distinct prognosis groups by the Elston and Ellis modification of the Scarff‐Bloom‐Richardson grading system: grade I/II (well‐differentiated), and III (poorly differentiated), indicating a low/intermediate and high aggressiveness respectively.^9^ Out of the 60 patients, 31 (51.67%) were of grade III and displayed significantly reduced expression of *Dab1* compared with patients of grade I/II (Figure 1E, 1.419 ± 0.255 vs 0.529 ± 0.098, *P* < 0.001). Similarly, patients with lymph node metastasis (56.67%) were also inversely correlated with the expression of *Dab1* (Figure 1F, 0.355 ± 0.088 vs 1.152 ± 0.295, *P* < 0.001). The receiver operating characteristic (ROC) curve was calculated to characterize the role of *Dab1* expression in lymph node metastasis.^10^ The area under the curve (AUC) was 0.769, indicating *Dab1* a diagnostic marker (Figure 1G).

Univariate and multivariate analysis using binary logistic regression (backward) were performed to characterize the association between breast cancer aggressiveness and clinicopathologic factors, including age at diagnosis, tumour volume, menopause status, ER, PR, HER2, p53, Ki67, lymph node state and the expression of *Dab1*. Results from the univariate analysis showed a negative correlation between the expression of *Dab1* and tumour differentiation grade (less Dab1 in aggressive grade III tumour) (Table S3, upper part; odds ratio (OR) = 4.644, *P* = 0.009). Both the univariate and multivariate analysis indicated a significant negative correlation between *Dab1* and lymph node metastasis (Table S3, lower part; OR = 6.111, *P* = 0.002 for univariate, OR = 3.991, *P* = 0.033 for multivariate), which suggested Dab1 was a potential molecular marker of clinical breast cancer progression.

### 3.2 Dab1 regulated cell apoptosis by regulating NF‐κB/Bcl2/caspase‐9

**Figure 2:**
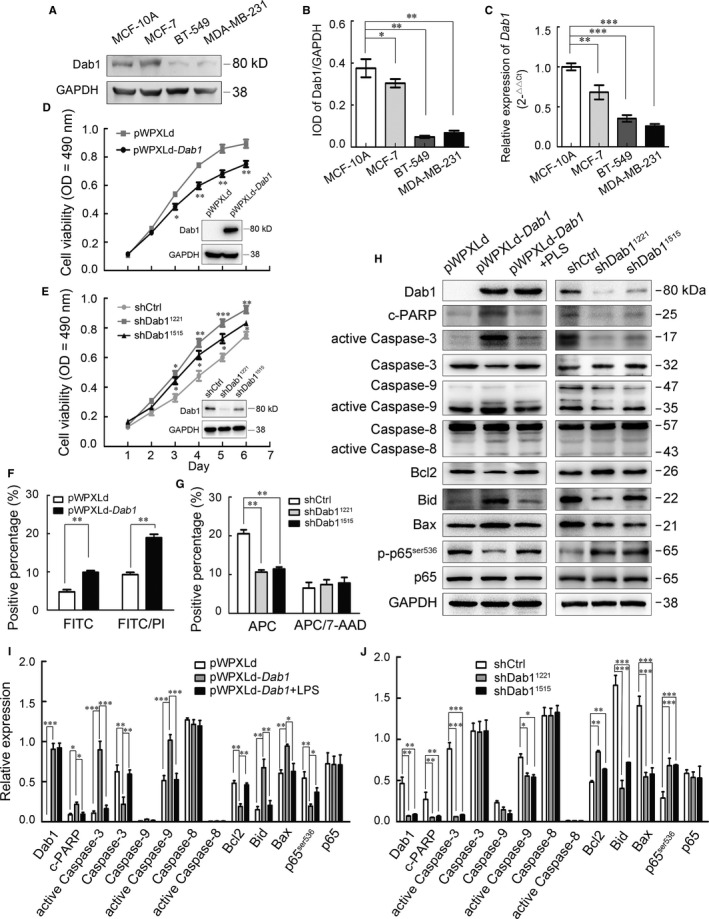
Dab1 in breast cancer cell lines was reduced and regulated cell apoptosis through NF‐κB/Bcl2/caspase‐9. (A) Western blot evaluation of the expression of Dab1 protein in MCF‐10A, MCF‐7, BT‐549 and MDA‐MB‐231. (B) Quantitative expression of the Dab1 in (A) using Gel‐Pro Analyzer 4 software. (C) Real‐time PCR detection of *Dab1* mRNA in these four cell lines. Relative expression of *Dab1* in MCF‐10A was normalized to 1, GAPDH was an internal control. (D) Cell viability was assessed with CCK8 assay in MDA‐MB‐231 infected with pWPXLd or pWPXLd‐*Dab1*. Insert is the Western blot evaluation of the expression of pWPXLd‐Dab1 in MDA‐MB‐231 cells. (E) The CCK8 assay was carried out to evaluate cell viability after Dab1 silencing. Insert is the Western blot analysed the silencing efficiency of shDab1^1221^ and shDab1^1515^. (F) Annexin V‐FITC/PI labelling was used to analyse the apoptosis of MDA‐MB‐231 cells transfected with pWPXLd or pWPXLd‐*Dab1*. (G) Annexin V‐APC/7‐AAD was used to assess the apoptosis of MCF‐10A infected with shDab1^1221^ or shDab1^1515^. (H) Western blot analysis of the expression of apoptosis related proteins in MDA‐MB‐231 transfected with pWPXLd, pWPXLd*‐Dab1* and pWPXLd*‐Dab1* treated with 10 μg/mL LPS or MCF‐10A infected with shCtrl, shDab1^1221^ or shDab1^1515^. (I, J) Quantitative analysis of expressed proteins in (H) with Gel‐Pro Analyzer 4 software. Data were shown as mean ± *SEM*. For all statistical analysis in this figure, *P < 0.05, **P < 0.01; ***P < 0.001

The reduced expression of Dab1 was also confirmed in breast cancer cells. Compared with the abundant expression in non‐tumourigenic human mammary epithelial cells MCF‐10A, Dab1 was slightly decreased in tumourigenic MCF‐7, significantly reduced in invasive cells BT‐549 and MDA‐MB‐231 (Figure 2A‐C). A recombinant *Dab1* based on lentiviral vector pWPXLd was cloned and transfected into MDA‐MB‐231 (Figure 2D, Insert). CCK8 analysis showed overexpression of Dab1 caused a significant reduction in cell survival (Figure 2D). On the other hand, lentivirus‐delivered shDab1^1221^ and shDab1^1515^ in MCF‐10A showed about 80% and 50% reduction of Dab1, respectively (Figure 2E, Insert), and resulted in the promotion of cell survival, especially in the shDab1^1221^ transfected cell (Figure 2E).

As cell survival can be affected both by cell proliferation and cell apoptosis, we next distinguished the role of Dab1 in these two processes. Results showed that neither MDA‐MB‐231 re‐expressing exogenous Dab1 nor MCF‐10A silenced by shDab1s showed any change in cell proliferation labelled by BrdU incorporation (Figure S1B‐E). However, Annexin V/DNA detection found that exogenous Dab1 promoted cell apoptosis in MDA‐MB‐231 (Figure 2F, Figure S1F), while Dab1 silencing resulted in decreased cell apoptosis in MCF‐10A cells (Figure 2G, Figure S1G).

Active caspase‐3 and its substrate, cleaved PARP (c‐PARP), both of which are hallmarks of cell apoptosis,^11^ were elevated in pWPXLd‐*Dab1* transfected MDA‐MB‐231 cells (Figure 2H,I). However, both of them were decreased in MCF‐10A transfected with shDab1^1221^ or shDab1^1515^ (Figure 2H,J). Caspase‐9 is an essential element in the intrinsic pathway of apoptosis, while Caspase‐8 is required in the extrinsic apoptosis pathway.^12^ To determine which pathway Dab1 was involved in cell apoptosis, the level of active caspase‐9 and caspase‐8 was evaluated by Western blot. Results showed that overexpression of Dab1 in MDA‐MB‐231 increased the active caspase‐9, while the silence of Dab1 in MCF‐10A reduced its expression. No change was observed in the expression of active caspase‐8 (Figure 2H‐J). Bcl2 family was reported to be crucial in regulating the intrinsic apoptosis pathway,^13^ and we found decreased Bcl2 and increased Bax and Bid in MDA‐MB‐231 expressing exogenous Dab1. Accordingly, Bcl2 was increased while Bax and Bid were reduced in MCF‐10A with shDab1^1221^ and shDab1^1515^ (Figure 2H‐J). NF‐κB is a transcription factor involved in cell apoptosis, and its increased transcription resulted in reduced apoptosis. The p65/p50 heterodimer is particularly relevant in regulating the expression of apoptotic proteins, such as anti‐apoptotic protein Bcl2.^14^ Here, we found NF‐κB p65^ser536^ phosphorylation, a well‐known active marker for NF‐κB,^15^ was reduced in MDA‐MB‐231 transfected with exogenous *Dab1*, while significantly increased in MCF‐10A with shDab1^1221^ and shDab1^1515^ (Figure 2H‐J). Lipopolysaccharide (LPS), which can induce the degradation of IκB and the translocation of p65/50 into the nucleus,^16^ was used to treat MDA‐MB‐231 (10 μg/mL) with exogenous Dab1. Western blot showed that the reduced p‐p65^ser536^ and Bcl2 expression were efficiently reverted by LPS treatment. Moreover, the active caspase‐9, active caspase‐3 and c‐PARP were all decreased, indicating that the artificial activation of NF‐κB reversed the Dab1 driven apoptosis (Figure 2H‐J), and the promoted cell apoptosis is indeed induced by Dab1.

Dab1 has been reported to be strongly down‐regulated in glioblastoma^17^ and neuroblastoma.^18^ In breast cancer, McAvoy has firstly reported the down‐regulation of Dab1, and overexpression of Dab1 resulted in decreased cell survival.^6^ Here, according to our study, the inhibited cell survival caused by Dab1 was resulted from its pro‐apoptotic function through regulating NF‐κB/Bcl2/caspase‐9 pathway. Combined with the clinical correlation between Dab1 and poor prognosis factors, these results suggested that *Dab1*, located in CFS, could also function as a suppressor gene through promoting cell apoptosis in breast cancer. Considering the un‐fulfilment of breast cancer treatment in the clinic, our identification of *Dab1* as a potential maker and suppressor in clinical breast cancer may benefit our understanding of pathogenesis and treatment of breast cancer.

## CONFLICT OF INTEREST

The authors confirm that there are no conflicts of interest.

## AUTHOR CONTRIBUTION

R‐JC, KL and W‐YX performed the main experiments and summarized the results. QL and SD analysed the data. R‐JC wrote the manuscript. X‐JZ and S‐SC provided the supervision and comments. All authors read and approved the final manuscript.
