# Supplementary figures and images for: Disabled‐1 is down‐regulated in clinical breast cancer and regulates cell apoptosis through NF‐κB/Bcl‐2/caspase‐9
Source: J Cell Mol Med. 2018 Nov 28;23(2):1622–7. doi: 10.1111/jcmm.14047 (PMC6349202; doi:10.1111/jcmm.14047)

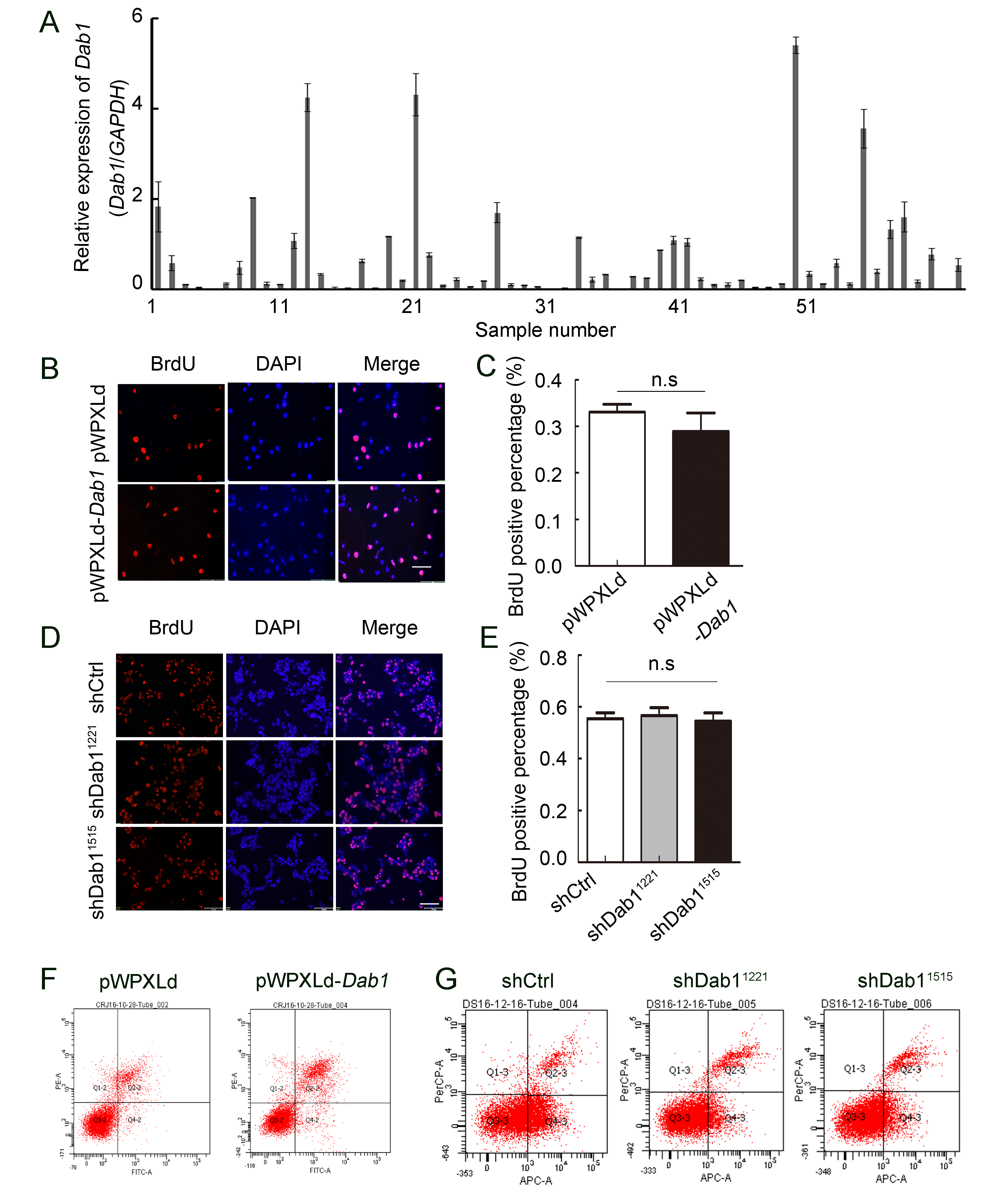

Supplement: Supplementary file 1 [file JCMM-23-1622-s001.tif]
