# Supplementary material for: Disabled‐1 is down‐regulated in clinical breast cancer and regulates cell apoptosis through NF‐κB/Bcl‐2/caspase‐9
Source: J Cell Mol Med. 2018 Nov 28;23(2):1622–7. doi: 10.1111/jcmm.14047 (PMC6349202; doi:10.1111/jcmm.14047)
Supplement: Supplementary file 2 [file JCMM-23-1622-s002.doc]

**Supplementary Methods and Materials**

**Antibodies**

The anti-Dab1 antibody was purchased from Millipore (AB5840, 1:1000). Mouse anti-GAPDH antibody was obtained from TransGene Biotech (HC301-01, 1:2000). Rabbit anti-cleaved-PARP (ab32064, 1:100000), rabbit anti-active caspase-3 (ab2302, 1:200), rabbit anti-Bcl2 (ab32124, 1:1000), rabbit anti-Bid (ab32060, 1:1000), rabbit anti-Bax (ab32503, 1:1000) and rat anti-BrdU (ab6326, 1:500) were purchased from Abcam. Rabbit anti-caspase-9 (#9502, 1:1000), rabbit anti-p-p65 Ser536 (#3033, 1:1000) and mouse anti-p65 (#6956, 1:1000) were from Cell Signaling Technology. Rabbit anti-caspase-3 was obtained from Millipore (ab1899, 1:200). Alexa Fluor 546-coupled secondary antibodies and HRP-conjugated secondary antibodies for rabbit or mouse IgG were from Invitrogen. DAPI was purchased from Sigma-Aldrich.

**Immunohistochemistry**

Paraffin-embedded tissue sections were subjected to the removal of paraffin with xylene, and rehydration with ethanol. Antigen retrieval was performed in 10 mM citrate buffer (pH = 6.0) with a temperature of 120 °C and pressure between 0.10 to 0.12 MPa for 2 min. After that, the sections were immersed in 3% H_2_O_2_ for 10 min to block endogenous peroxidase activity, followed by the incubation of primary antibody and then appropriate secondary antibody according to the UltraSensitiveTM SP (Mouse/Rabbit) IHC Kit (KIT-9710, MAB, Fuzhou, China). To show the expression of the antigen, peroxidase-based DAB kit (DAB-0031, MAB, Fuzhou, China) was used, having the nuclei counterstained with hematoxylin. Images were captured with a Nikon microscope (Eclipse E200, Tokyo, Japan).

**Cell lines and cell culture**

MCF-10A cells were cultured in DMEM/F12 containing 5% horse serum, 20 ng/mL EGF, 0.5 µg/mL hydrocortisone, 100 ng/mL cholera toxin, 10 µg/mL insulin and penicillin/streptomycin. MCF-7 and BT-549 cells were grown in MEM or RPMI-1640 culture medium respectively supplemented with 10% fetal bovine serum. MDA-MB-231 cells were cultured in Leibovitz’s L-15 containing 10% fetal bovine serum at 37ºC without CO_2_. 293T cells were grown in DMEM supplemented with 10% fetal bovine serum. To activate the NF-κB, MDA-MB-231 transfected with exogenous Dab1 were treated with 10 μg/mL Lipopolysaccharide[^1^](#_ENREF_1) (Cat. No. L6143, Sigma-Aldrich).

**Lysis and immunoblotting**

Cultured cells or fresh tumor tissues were washed twice with PBS and lysed in RIPA buffer (50 mM Tris-HCl, pH = 7.4, 5 mM EDTA, 150 mM NaCl, 1% Triton X-100 and 0.1% SDS) containing protease inhibitor cocktail (Cat. No. 11836153001, Roche) and 1 mM PMSF for 30 minutes on ice. Then the cell/tissue debris was removed by centrifugation at 12,000 g for 20 minutes at 4ºC. The protein concentration of the supernatant was measured with standard Bradford protein assay. 5× Laemmli buffer was added and boiled for 5 minutes. The protein samples were separated by SDS-PAGE and then transferred to the PVDF membranes (0.45 µm pore size, Cat. No. IPVH00010, Millipore). The membranes were blocked at room temperature in 5% BSA for 1 hour. The primary antibodies were diluted with 2% BSA in TBST (pH = 7.4) and incubated overnight at 4°C. After incubation, the membranes were washed in TBST for 30 minutes, followed by incubation of HRP-conjugated secondary antibodies for 1 hour at room temperature. Immunoreactive bands were detected with Tanon High-sig ECL Western Blotting Substrate (Cat. No.180-5001, Tanon, Shanghai, China). The bands were scanned by CCD system, and the greyscale images were analyzed by Gel-Pro Analyzer 4.0 software (Media Cybernetics, Bethesda, MD, US).

**RNA extraction and Real-time PCR analysis**

Total RNA was extracted from breast cancer cells or fresh tumor tissues using TriPure isolation reagent (Cat No. 11667165001, Roche). cDNA synthesis was performed using One-step gDNA removal and cDNA synthesis superMix (AT-311-03, TransGen Biotech, Beijing, China). Real-time PCR was performed using SYBR Premix Ex Taq^TM^ II (Cat No. RR820A, Takara, Dalian, China) with the ABI StepOnePlus ^TM^ Real-time PCR System. Primers used are as follows (5’ to 3’, sense, antisense):

*Dab1*, CTGTGAAAACCAGCGCCAAG, ACGTTTGCATAGCACCTTGAG;

*GAPDH,* ATGACCCCTTCATTGACCTCA, GAGATGATGACCCTTTTGGCT.

**Lentiviral packaging and transfection**

Target shRNAs were designed with BLOCK-iT™ RNAi Designer and target the region encompassing nucleotides 1221-1239 (Dab1^1221^) or 1515-1533 (Dab1^1515^) in the human *Dab1* gene. The scrambled shRNA, TTCTCCGAACGTGTCACGT, was designed as a nonspecific control. The shRNAs were synthesized and inserted into lentiviral plasmid PLL3.7. The *Dab1* expressing plasmid constructed before[^2^](#_ENREF_2) was cloned into the *Pme*I and *Sma*I sites of a lentivirus expressing vector pWPXLd (#12257, Addgene) by PCR with following primers:

Forward: GGGTTTAAACTAATGTCAACTGAGACAG;

Reverse: TCCCCCGGGCTAGCTACCGTCTTGTGGACTT.

For lentiviral packaging, shRNAs including Dab1 shRNAs and scrambled shRNA, or Dab1 expressing vector pWPXLd-*Dab1* (pWPXLd as control) were transferred with the packaging plasmid psPAX2 (#12260, Addgene) and the envelop plasmid pMD2.G (#12259, Addgene), with a ratio of 2:1:1 into the packaging cell line 293T. Viral supernatants were harvested 48 hours and 72 hours after transfection, filtered with 0.45 μm filters (Millipore) and concentrated using Amicon Ultra centrifugal filters (100KD MWCO, UFC910008, Millipore). The concentrated virus was used to infect MCF-10A or MDA-MB-231, respectively.

**Cell viability assay**

Cell growth was analyzed by CCK8 (cell counting kit-8, #CK04, Dojindo Molecular Technologies) assay. Cells were seeded into 96-well plates, with the number of 4000 per well for MCF-10A or 3000 per well for MDA-MB-231. After the incubation of indicated time, 10 µL of the CCK8 solution was added to each well and incubated for additional 2~3 hours at 37°C. Then cell viability was determined by detecting the absorbance values at 450 nm.

**BrdU incorporation assay**

Cells were grown on the coverslips in the 12-well plates. BrdU (10 µM) was added into MCF-10A cells or MDA-MB-231 cells. 8 hours later, cells were fixed with 4% PFA, permeabilized with 1% Triton X-100 and denatured by 1M HCl. BrdU primary antibody and Alexa Fluor 546-secondary antibody were used to label cells. Images were captured with Olympus FV1000 laser scanning confocal microscope.

**Apoptosis assay**

MDA-MB-231 infected by pWPXLd or pWPXLd-*Dab1* lentivirus were labeled with Annexin V-FITC kit following the manufacturer recommendations. Briefly, cells were harvested and 1×10^5^ cells were suspended in binding buffer. Annexin V-FITC and propidium iodide were added to label the apoptotic cells for 10min in dark, following with analysis by Flow Cytometry. As the expression of GFP in MCF-10A cells with shCtrl, shDab1^1221^ or shDab1^1515^ became stable, Annexin V-APC/7-AAD kit was used to analyze apoptosis following the same protocols.

**Statistical analysis**

Statistical analysis was performed with SPSS17.0 (Chicago, IL, USA). All data were presented as Means ± *SEM*. Kruskal-Wallis test was used for the comparisons of three or more groups, and non-parametric Mann-Whitney *U*-test was used for the comparisons between two groups. Receiver operating characteristic (ROC) curve was used to assess the association of *Dab1* with lymph node metastasis. Univariate and multivariate logistic regressions (backward) were performed to analyze the correlation between decreased expression of *Dab1* and tumor differentiation grade or lymph node metastasis in clinical specimens. All Dab1 IOD values were analyzed by Image-Pro Plus (Media Cybernetics, USA) and listed in S1 Table. All the graphics were performed using GraphPad Prism 5.0. *P* value < 0.05 was considered as significant.

**Reference**

1. Pidgeon GP, Barr MP, Harmey JH, et al. Vascular endothelial growth factor (VEGF) upregulates BCL-2 and inhibits apoptosis in human and murine mammary adenocarcinoma cells. Br J Cancer. 2001; 85: 273-8.

2. Zhang JH, Zhao YF, He XX, et al. DCC-Mediated Dab1 Phosphorylation Participates in the Multipolar-to-Bipolar Transition of Migrating Neurons. Cell Rep. 2018; 22: 3598-611.

**Table S1. The Clinical pathologic information of the breast cancer paraffin sections (January 2016 – November** 2016).

| No. | Age | Latency | Size (mm) | Lymph  state | Grade | Ki67 | IOD of Dab1 in tumor | IOD of Dab1 in adjacent |
| --- | --- | --- | --- | --- | --- | --- | --- | --- |
| 1 | 52 | 1y | 10×8×6 | 0/21 | I | 3%+ | 3793.093 | 5659.032 |
| 2 | 43 | 1m | 21×14×7 | 1/21 | II | 20%+ | 4098.765 | 10934.632 |
| 3 | 51 | 4m | 24×21×18 | 3/22 | III | 50%+ | 567.832 | 8201.213 |
| 4 | 42 | 6m | 31×25×12 | 3/18 | III | 50%+ | 984.012 | 6589.089 |
| 5 | 66 | 1m | 18×15×12 | 0/24 | I | <1%+ | 4730.132 | 9112.039 |
| 6 | 52 | 2m | 10×10×8 | 0/20 | II | 5%+ | 5320.094 | 5804.381 |
| 7 | 68 | 5m | 25×15×10 | 1/18 | II | 20%+ | 4392.879 | 7450.978 |
| 8 | 72 | 6m | 40×30×30 | 5/25 | III | 60%+ | 357.561 | 8342.094 |
| 9 | 61 | 1y | 15×10×6 | 0/24 | I | 3%+ | 3987.456 | 9834.32 |
| 10 | 52 | 2m | 20×15×15 | 2/22 | II | 15%+ | 3781.237 | 8334.278 |
| 11 | 45 | 3m | 55×25×25 | 3/24 | III | 40%+ | 543.276 | 9112.374 |
| 12 | 47 | 4m | 22×18×16 | 1/16 | III | 50%+ | 790.344 | 6459.782 |
| 13 | 65 | 5m | 15×12×10 | 0/21 | I | <1%+ | 5312.247 | 5543.232 |
| 14 | 43 | 6m | 50×40×20 | 0/22 | II | 5%+ | 3109.332 | 7303.978 |
| 15 | 70 | 2m | 15×15×15 | 2/15 | III | 60%+ | 1208.654 | 8317.249 |
| 16 | 68 | 3m | 50×45×35 | 4/25 | III | 50%+ | 1002.366 | 7231.38 |
| 17 | 55 | 1y | 10×10×6 | 0/25 | I | 3%+ | 1876.065 | 9942.336 |
| 18 | 53 | 1m | 25×18×15 | 3/20 | III | 60%+ | 990.675 | 7895.468 |
| 19 | 39 | 2m | 30×25×15 | 4/18 | III | 40%+ | 1102.379 | 8623.946 |
| 20 | 49 | 3m | 22×16×16 | 4/22 | III | 60%+ | 866.754 | 7632.089 |
| 21 | 71 | 6m | 40×30×10 | 1/25 | III | 50%+ | 343.688 | 6321.638 |
| 22 | 72 | 3m | 35×30×20 | 3/22 | II | 25%+ | 1546.790 | 6780.341 |
| 23 | 46 | 3m | 25×10×10 | 2/16 | II | 30%+ | 2376.858 | 7079.943 |
| 24 | 53 | 1y | 15×8×8 | 340/20 | II | 5%+ | 3356.347 | 7335.468 |
| 25 | 43 | 2m | 50×30×20 | 2/16 | III | 60%+ | 579.355 | 6998.468 |
| 26 | 57 | 1m | 30×20×10 | 3/20 | III | 45%+ | 780.342 | 8327.056 |
| 27 | 70 | 3m | 35×25×15 | 2/25 | III | 40%+ | 1214.897 | 7390.611 |
| 28 | 48 | 5m | 25×20×20 | 3/18 | III | 50%+ | 409.806 | 8814.369 |
| 29 | 62 | 3m | 20×10×10 | 2/20 | II | 30%+ | 1397.407 | 6903.467 |
| 30 | 55 | 2m | 10×10×5 | 0/21 | I | <1%+ | 4310.668 | 7880.565 |
| 31 | 46 | 3m | 18×12×12 | 0/22 | II | 20%+ | 1547.034 | 8453.899 |
| 32 | 57 | 4m | 28×18×15 | 2/18 | II | 30%+ | 2875.09 | 7889.456 |
| 33 | 48 | 2m | 35×20×15 | 1/16 | II | 20%+ | 986.004 | 6709.352 |
| 34 | 53 | 1m | 30×15×15 | 0/20 | III | 50% | 695.487 | 6394.899 |
| 35 | 59 | 4m | 30×20×10 | 3/18 | III | 80% | 599.675 | 8980.697 |
| 36 | 39 | 3m | 15×12×12 | 2/25 | III | 30% | 669.876 | 6998.488 |
| 37 | 47 | 1y | 50×25×25 | 4/25 | III | 50% | 1359.35 | 8987.669 |
| 38 | 59 | 6m | 25×15×15 | 0/16 | II | 30% | 3265.843 | 9546.774 |

**Table S2. Clinic pathologic information and *Dab1*** expression profile in tumor tissues compared to paired surrounding tissues in 60 patients (January 2017 - July 2017).

| No. | Age | Latency | Size (mm) | Lymph  state | Grade | Menses  (N/M) | ER | PR | HER2 | p53 | Ki67 | Dab1  2^-△△Ct^  (Mean±SE) |
| --- | --- | --- | --- | --- | --- | --- | --- | --- | --- | --- | --- | --- |
| 1 | 61 | 1y | 45×30×25 | 0/24 | II | M | 90%, 3+ | 80%, 3+ | - | - | 15%+ | 1.831±0.553 |
| 2 | 46 | 1m | 10×8×5  8×7×6 | 0/20 | II | N | 30%, 2+ | 5%, 3+ | - | - | 10%+ | 0.589±0.167 |
| 3 | 54 | 2m | 35×25×20 | 4/25 | III | M | 90%, 3+ | 10%, 3+ | - | - | 20%+ | 0.119±0.001 |
| 4 | 39 | 3m | 12×9×8 | 4/17 | III | N | - | - | - | 50%+ | 50% | 0.051±0.003 |
| 5 | 73 | 5d | 20×18×10 | 1/25 | III | M | - | - | - | - | 60%+ | 0.018±0.001 |
| 6 | 43 | 6m | 50×40×20 | 0/22 | II | N | - | - | 2+ | - | <5% | 0.129±0.021 |
| 7 | 71 | 4m | 20×15×15 | 0/16 | II | M | 90%, 3+ | 80%, 2+ | - | - | 30%+ | 0.485±0.143 |
| 8 | 70 | 1y | 50×35×30 | 4/20 | III | M | - | - | 2+ | - | 60%+ | 2.02±0.008 |
| 9 | 57 | 3d | 18×16×12 | 1/32 | III | M | - | - | 3+ | - | 20% | 0.129±0.035 |
| 10 | 39 | 1m | 45×35×15 | 2/23 | III | N | - | - | 2+ | - | 30%+ | 0.113±0.006 |
| 11 | 49 | 1m | 28×25×20 | 0/17 | II | N | 40%, 3+ | 80%, 2+ | - | 10%+ | 10%+ | 1.072±0.161 |
| 12 | 50 | 1m | 18×15×15 | 0/18 | II | N | 80%, 3+ | 80%, 3+ | + | 80%+ | 40%+ | 4.247±0.309 |
| 13 | 64 | 7d | 15×10×15 | 6/18 | III | M | 3+ | - | - | - | 80%+ | 0.332±0.017 |
| 14 | 42 | 10d | 22×16×12 | 2/28 | III | N | - | - | 2+ | + | 20%+ | 0.034±0.018 |
| 15 | 60 | 2m | 25×20×13 | 1/18 | III | M | - | - | 2+ | 80%+ | 50%+ | 0.036±0.002 |
| 16 | 56 | 1y | 15×18×20 | 0/19 | II | M | 90%, 3+ | 60%, 2+ | - | 10%+ | 30%+ | 0.631±0.042 |
| 17 | 54 | 6m | 25×24×18 | 3/21 | III | M | 90%, 3+ | 90%, 3+ | - | - | 10%+ | 0.032±0.006 |
| 18 | 42 | 15d | 55×36×34 | 2/27 | III | N | 80%3+ | 90%3+ | - | + | 10%+ | 1.171±0.003 |
| 19 | 43 | 3m | 45×35×20 | 0/16 | III | N | 20% | 70%, 3+ | - | 80%, 3+ | 10%+ | 0.196±0.012 |
| 20 | 48 | 2m | 20×20×15 | 0/18 | I | N | 60%, 3+ | 90%, 3+ | - | 80%3+ | 10%+ | 4.31±0.473 |
| 21 | 62 | 5d | 60×50×20 | 0/31 | II | M | 80%, 3+ | 10%+ | - | - | 15%+ | 0.765±0.054 |
| 22 | 43 | 15d | 40×35×25 | 1/20 | III | N | - | - | - | - | 30%+ | 0.089±0.018 |
| 23 | 30 | 3m | 35×15×15 | 0/19 | II | N | 90%, 3+ | 2%+ | - | 1%+ | 5%+ | 0.220±0.031 |
| 24 | 44 | 2m | 20×15×15 | 5/23 | III | N | 80%, 3+ | 60%, 3+ | - | 20%+ | 60%+ | 0.062±0.007 |
| 25 | 50 | 2m | 25×20×15 | 1/34 | III | M | - | - | - | 90% | 60% | 0.191±0.009 |
| 26 | 46 | 5m | 30×20×20 | 0/20 | Ⅲ | N | - | - | - | 80%+ | 20%+ | 1.7±0.219 |
| 27 | 47 | 1m | 22×18×12 | 0/17 | Ⅲ | N | 90%,2+ | - | - | 80%+ | 60%+ | 0.11±0.026 |
| 28 | 44 | 2m | 12×11×10 | 0/16 | II | M | - | - | - | 80% | 5% | 0.103±0.003 |
| 29 | 42 | 5m | 18×15×10 | 1/18 | Ⅲ | N | - | - | - | 80%+ | 40%+ | 0.066±0.003 |
| 30 | 51 | 10d | 35×20×20 | 5/16 | Ⅲ | M | - | - | - | - | 30%+ | 0.019±0.002 |
| 31 | 46 | 1m | 35×35×32 | 2/19 | Ⅲ | N | - | - | - |  | 50%+ | 0.029±0.002 |
| 32 | 59 | 6m | 50×45×40 | 2/25 | Ⅲ | M | - | - | - | - | 50%+ | 1.149±0.015 |
| 33 | 58 | 1m | 12×12×10 | 2/24 | Ⅲ | M | - | - | - | 30%+ | 70%+ | 0.220±0.059 |
| 34 | 30 | 10d | 80×60×40 | 0/38 | Ⅲ | N | - | - | - | 90%+ | 30%+ | 0.336±0.005 |
| 35 | 75 | 2m | 50×48×38 | 17/18 | Ⅲ | M | - | - | - | - | 30%+ | 0.014±0.003 |
| 36 | 44 | 15d | 20×15×15 | 1/22 | II | N | - | - | - | - | 40%+ | 0.283±0.01 |
| 37 | 59 | 2m | 37×15×5 | 5/20 | II | M | - | - | - | - | <5%+ | 0.244±0.09 |
| 38 | 57 | 7d | 18×15×12 | 0/26 | Ⅲ | M | - | - | 3+ | 80%+ | 20%+ | 0.865±0.06 |
| 39 | 55 | 3m | 45×30×25 | 17/22 | Ⅲ | M | - | - | 2+ | - | 40%+ | 1.09±0.08 |
| 40 | 50 | 2m | 7×5×7 | 2/18 | Ⅲ | M | - | - | 2+ | - | 30%+ | 1.04±0.09 |
| 41 | 27 | 3m | 40×40×30 | 0/1 | II | N | - | - | 2+ | 90%+ | 30%+ | 0.227±0.036 |
| 42 | 59 | 2y | 40×35×30 | 12/20 | II | M | - | - | 2+ | - | 30%+ | 0.105±0.017 |
| 43 | 51 | 6m | 50×30×30 | 12/19 | II | M | - | - | 2+ | - | 10%+ | 0.110±0.03 |
| 44 | 56 | 1y | 15×15×7 | 0/20 | II | M | - | - | 3+ | 1%+ | 20%+ | 0.207±0.008 |
| 45 | 41 | 3y | 50×40×25 | 18/21 | Ⅲ | N | - | - | 2+ | 80%+ | 20%+ | 0.052±0.006 |
| 46 | 61 | 3m | 20×20×10 | 7/17 | Ⅲ | M | - | - | 2+ | 90%+ | 30%+ | 0.045±0.007 |
| 47 | 53 | 2y | 20×10×10 | 0/17 | II | M | 90%3+ | 10%2+ | 2+ | <1%+ | 50%+ | 0.121±0.006 |
| 48 | 49 | 1m | 18×12×11 | 0/23 | II | N | 90%3+ | 5%2+ | 2+ | <1%+ | 30%+ | 5.4±0.987 |
| 49 | 36 | 2y | 25×20×18 | 0/26 | II | N | 80%3+ | 80%3+ | 2+ | 90%+ | 20%+ | 0.346±0.051 |
| 50 | 47 | 3y | 60×45×25 | 9/21 | II | N | 70%2+ | 30%3+ | 2+ | 5%+ | 10% | 0.134±0.000 |
| 51 | 46 | 1y | 35×30×25 | 0/25 | II | N | 70%3+ | 80%3+ | - | + | 10%+ | 0.577±0.086 |
| 52 | 45 | 2y | 20×15×10 | 1/17 | II | N | 50%2+ | 30%2+ | - | - | 20%+ | 0.129±0.023 |
| 53 | 52 | 1y | 25×20×20 | 0/23 | II | M | 80%+ | 80%+ | - | <1%+ | 10%+ | 3.557±0.425 |
| 54 | 46 | 6y | 15×15×12 | 0/17 | II | N | 30%2+ | 50%3+ | 2+ | 90%+ | 5%+ | 0.399±0.041 |
| 55 | 48 | 7m | 20×15×15 | 0/23 | II | N | - | - | 2+ | 60%+ | 30%+ | 1.329±0.192 |
| 56 | 52 | 20y | 22×15×15 | 1/21 | II | M | 3%1+ | 80%2+ | 2+ | 70%+ | 40%+ | 1.604±0.337 |
| 57 | 61 | 4y | 30×25×15 | 0/21 | II | M | 80%2+ | 90%3+ | - | - | 20%+ | 0.177±0.028 |
| 58 | 60 | 4y | 20×20×18 | 1/22 | II | M | 50%2+ | 30%1+ | - | - | 20%+ | 0.779±0.126 |
| 59 | 51 | 4m | 12×10×10 | 1/21 | II | N | 80%3+ | 60%2+ | - | - | <5%+ | 0.019±0.000 |
| 60 | 39 | 3m | 15×15×10 | 1/17 | II | N | 40%2+ | 80%3+ | - | - | 5%+ | 0.540±0.147 |

**Table S3. *Dab1* expression associated with breast cancer aggressiveness (tumor differentiation grade) and lymph node metastasis^#^**

|  | Univariate | | |  | Multivariate | | | |
| --- | --- | --- | --- | --- | --- | --- | --- | --- |
|  |  | |  |  |  | 95% confidence level | |  |
|  | Odd ratio | | P value |  | Odd ratio | Lower | Upper | P value |
| **Tumor differentiation grade I/II (well-differentiated, reference) versus grade III (poorly differentiated or undifferentiated)** | | | | | | | | |
| Age (T1 Stage as reference) | | 0.661 | 0.576 |  |  |  |  |  |
| Tumor volume (≤ 20mm as reference) | | 2.027 | 0.203 |  |  |  |  |  |
| Non-menopause (menopause as reference) | | 1.495 | 0.606 |  |  |  |  |  |
| Estrogen receptor positive (negative as reference) | | 0.13 | 0.000 |  |  |  |  |  |
| HER2 positive (negative as reference) | | 0.729 | 0.603 |  |  |  |  |  |
| Progesterone receptor positive (negative as reference) | | 0.085 | 0.000 |  |  |  |  |  |
| p53 (negative as reference) | | 0.882 | 1.000 |  |  |  |  |  |
| Ki67 (≤ 20% positive as reference) | | 4.04 | 0.011 |  | 3.969 | 1.092 | 14.424 | 0.036 |
| Positive lymph node metastasis (negative as reference) | | 10.080 | 0.000 |  | 6.220 | 1.636 | 23.649 | 0.007 |
| *Dab1* in tumor tissue (< 50% decrease as reference) | | 4.644 | 0.009 |  | 3.140 | 0.834 | 11.817 | 0.091 |
| **Negative lymph node metastasis (N0, reference) versus positive (N+)** | | | | | | | | |
| Age (T1 Stage as reference) | | 0.770 | 0.779 |  |  |  |  |  |
| Tumor volume (≤ 20mm as reference) | | 1.224 | 0.795 |  |  |  |  |  |
| Non-menopause (menopause as reference) | | 2.286 | 0.192 |  |  |  |  |  |
| Estrogen receptor positive (negative as reference) | | 0.213 | 0.000 |  |  |  |  |  |
| HER2 positive (negative as reference) | | 0.990 | 1.000 |  |  |  |  |  |
| Progesterone receptor positive (negative as reference) | | 0.221 | 0.009 |  |  |  |  |  |
| Ki67 (≤ 20% positive as reference) | | 2.585 | 0.117 |  |  |  |  |  |
| p53 (negative as reference) | | 0.164 | 0.001 |  |  |  |  |  |
| Tumor differentiation grade III (grade I/II as reference) | | 10.08 | 0.000 |  | 7.439 | 2.073 | 26.703 | 0.002 |
| *Dab1* in tumor tissue (< 50% decrease as reference) | | 6.111 | 0.002 |  | 3.993 | 1.119 | 14.253 | 0.033 |

^#^, Binary Logistic regression (backward) in 60 breast cancer specimens. Triple repeated per specimen.

**Figure legends**

**Figure S1. Dab1 does not affect the proliferation of breast cancer cells.** (A) Real-time PCR was used to examine the expression of *Dab1* mRNA in breast cancer specimens from 60 patients. *Dab1* in adjacent tissue was normalized to 1, and *Dab1* in breast cancer tissues were related to each pairing adjacent tissues. (B, D) BrdU incorporation analysis was used to assay the proliferation of MDA-MB-231 transfected with pWPXLd or pWPXLd-*Dab1*, as well as MCF-10A infected by shCtrl, shDab11221 or shDab11515. (C, E) Statistic analysis of BrdU positive percentage. (F, G) MDA-MB-231 were labeled by Annexin V-FITC/PI (F), and MCF-10A were labeled with Annexin V-APC/7-AAD (G). Flow cytometry was used to investigate cell apoptosis.
